# A Rapid LC–MS/MS Method for Quantification of Biogenic Amines in Meat: Validation and Application for Food Safety Monitoring

**DOI:** 10.3390/mps8050106

**Published:** 2025-09-10

**Authors:** Giulia Rampazzo, Giacomo Depau, Giampiero Pagliuca, Elisa Zironi, Andrea Serraino, Federica Savini, Teresa Gazzotti

**Affiliations:** Department of Veterinary Medical Science, Alma Mater Studiorum University of Bologna, Via Tolara di Sopra 50, Ozzano dell’Emilia, 40064 Bologna, Italy; giulia.rampazzo4@unibo.it (G.R.); giacomo.depau2@unibo.it (G.D.); elisa.zironi@unibo.it (E.Z.); andrea.serraino@unibo.it (A.S.); federica.savini3@unibo.it (F.S.); teresa.gazzotti@unibo.it (T.G.)

**Keywords:** biogenic amines, LC-MS/MS, meat, quality control, food safety

## Abstract

Biogenic amines (BAs) are nitrogenous compounds naturally present in protein-rich foods, whose accumulation may indicate spoilage and pose health risks. This study presents the development and validation of a rapid LC–MS/MS method for the simultaneous quantification of six BAs—putrescine (PUT), cadaverine (CAD), histamine (HIS), tyramine (TYR), spermidine (SPD), and spermine (SPM)—in meat products, without requiring derivatisation. Sample preparation was optimized to enhance extraction efficiency and reproducibility, using 0.5 M HCl and a double-centrifugation protocol to avoid matrix interference. Chromatographic separation was optimized using a C18 column and acidified ammonium formate/acetonitrile mobile phases. The method showed good linearity (R^2^ > 0.99), trueness between −20% and +20%, and acceptable precision (RSD_r_ and RSD_R_ ≤ 25%). Limits of quantification were established at 10 µg/g for all analytes. The method was applied to ten commercial meat samples, where PUT, TYR, and SPD were the most frequently detected amines. Although HIS and TYR levels were below toxicological thresholds for healthy individuals, one sample showed TYR levels potentially concerning for monoamine oxidase inhibitors -treated consumers. The Biogenic Amine Index (BAI) further supported product quality assessment, identifying early spoilage in selected cases. This method offers a rapid, robust and efficient tool for routine monitoring of BAs in meat products, supporting food safety and quality control initiatives.

## 1. Introduction

Biogenic amines (BAs) are low-molecular-weight nitrogenous compounds naturally present in many foods [1]. In humans, they play important physiological roles such as neurotransmission, regulation of growth and blood pressure, modulation of the intestinal immune system, antioxidant activity, and promotion of cell proliferation and differentiation [1]. Despite these functions, excessive intake, particularly from contaminated foods, can cause adverse effects on the nervous, respiratory, and cardiovascular systems, as well as allergic reactions. Symptoms range from headaches, palpitations, nausea, vomiting, diarrhea, urticaria, and blood pressure fluctuations to, in severe cases, death [2].

Histamine (HIS) and tyramine (TYR) are the most toxic BAs [3,4,5]. HIS poisoning (scombroid poisoning) can trigger severe symptoms within hours, while TYR intoxication is associated with headaches, nausea, palpitations, and hypertension [1]. Cadaverine (CAD) and putrescine (PUT), although less toxic, can inhibit enzymes involved in HIS and TYR metabolism, thereby enhancing their toxicity [5]. Several BAs—including spermidine (SPD), spermine (SPM), PUT, and CAD—can also react with nitrites to form carcinogenic nitrosamines [2]. BAs are found at variable levels in protein- and amino acid-rich foods such as fish, meat, dairy, wine, beer, vegetables, fruits, nuts, and chocolate [6,7]. Their accumulation is a marker of spoilage, reflecting poor hygienic quality. Formation depends on raw material composition, microbial contamination, processing hygiene, storage conditions, fermentation, ripening, and packaging [8,9]. Importantly, BAs are thermostable and persist after cooking or pasteurization [1,10]. To assess meat and fish quality, the Biogenic Amine Index (BAI) has been proposed, where values below 1 indicate good quality and values above 10 suggest spoilage [6].

Meat is particularly prone to BA accumulation due to its high protein content and proteolytic activity during storage or processing [1,3]. Fresh meat naturally contains SPD and SPM (≈10–60 mg/kg), but levels of HIS, CAD, PUT, and TYR can increase during storage through proteolysis [11,12]. A CAD and PUT increase >15 mg/kg in raw pork has been suggested as a spoilage indicator [13]. Recently, dry-aged meat has gained popularity for its intense flavor and tenderness. However, the maturation environment (nutrient-rich, suitable pH, high water activity) favors microbial growth, including decarboxylating bacteria responsible for the formation of BAs [14,15,16]. As a result, BA levels may reach concentrations posing health risks, especially for sensitive consumers. This underlines the importance of reliable analytical methods for BA detection in such products.

BA analysis is essential for ensuring food quality and safety. Various analytical methods exist for their determination in food [3,5,8,17,18]. Commonly, analytical protocols involve extraction with acids or organic solvents [19,20] followed by separation via high-performance liquid chromatography (HPLC), gas chromatography (GC), thin-layer chromatography (TLC) and capillary electrophoresis (CE), often coupled with fluorescence detection (FLD), ultraviolet (UV), diode array detection (DAD), or mass spectrometry (MS) [21,22]. Since BAs have poor native absorbance, derivatization (commonly with dansyl chloride) is widely used to enhance detection [22]. However, derivatization can be labor-intensive, requiring precise control of reaction conditions, long incubation times, heating, and additional clean-up steps—factors that can significantly increase the overall complexity and duration of the analysis. New approaches, such as biosensors and flow injection analysis, are being explored for rapid on-site detection [23].

Given their toxicological importance and persistence in food, developing fast, sensitive, and user-friendly methods is a priority. In this context, the present study proposes a rapid liquid chromatography coupled with tandem-mass spectrometry (LC-MS/MS) method for BA determination in meat samples without derivatization. The method was fully validated and applied to real matrices.

## 2. Materials and Methods

### 2.1. Reagents and Chemicals

BAs analytical standards: HIS, HIS-d4 dihydrochloride (internal standard IS), CAD dihydrochloride, PUT, PUT-d4 dihydrochloride (IS), SPM, SPD, and TYR (standard purity ≥ 97.0%), were obtained from Sigma-Aldrich (St. Louis, MO, USA). High-performance solvents and reagents—including acetonitrile, methanol, hydrochloric acid (HCL) 37%, and formic acid—were sourced from Merck (Darmstadt, Germany), and ammonium formate (LC-MS grade) was also procured from Sigma-Aldrich. Ultrapure water was generated in-house using a purification system (Sartorius, Varedo, Italy). Filtration materials, specifically paper filters, were purchased from Waters Corporation (Milford, MA, USA).

### 2.2. Preparation of Standard Solutions

Single stock solutions were prepared by dissolving 50 mg of each standard in 5 mL of an 80:20 (*v*/*v*) methanol-water mixture and stored at −20 °C until use. Thus, appropriate amounts of stock solution were diluted with 80:20 (*v*/*v*) methanol-water to obtain a mixed standard solution at a concentration of 1000 μg/mL. For internal standard solutions, 10 mg of each IS were dissolved in separate glass flasks with 10 mL of 80:20 (*v*/*v*) methanol-water mixture and stored at −20 °C until use.

### 2.3. Sampling

A total of 10 raw beef samples, originating from different batches but with the same expire date, were purchased from local Italian markets. All samples were stored at −80 °C until homogenization.

### 2.4. Sample Preparation

One gram of minced muscle tissue was weighed into a 15 mL polypropylene centrifuge tube. 20 µL of each IS solution was added to the sample. Four milliliters of hydrochloric acid solution 0.5 M were added to the sample. The mixture was homogenized using an ultraturrax T25 homogenizer (IKA, Staufen, Germany) until complete dispersion was achieved. The homogenate was then centrifuged at 9000 rpm for 10 min at 4 °C. The resulting supernatant was carefully filtered through qualitative filter paper into a clean 15 mL polypropylene tube and subjected to a second centrifugation under the same conditions (9000 rpm, 4 °C, 5 min) to clarify the extract further.

An aliquot (100 µL) of the final supernatant was diluted 1:10 (*v*/*v*) with a mixture of acetonitrile containing 0.2% formic acid and water containing 2 mM ammonium formate and 0.2% formic acid (1:1, *v*/*v*). A second dilution (1:50, *v*/*v*) was performed using the same solvent composition. The diluted samples were analyzed by LC-MS/MS.

### 2.5. LC–MS/MS Conditions

LC–MS/MS analysis was performed using an ACQUITY Ultra Performance Liquid Chromatography (UPLC) system coupled to a Xevo TQ-S triple quadrupole mass spectrometer (Waters Corporation, Beverly, MA, USA). Chromatographic separation was achieved using a Waters Acquity UPLC^®^ BEH C18 column (2.1 mm × 50 mm, 1.7 µm particle size) equipped with UPLC^®^ BEH C18, 1.7 µm VanGuard Pre-column (Waters Corporation, Beverly, MA, USA).

The mobile phases consisted of phase A: water containing 2 mM ammonium formate and 0.2% formic acid (*v*/*v*), phase B: acetonitrile containing 0.2% formic acid (*v*/*v*).

The gradient elution program was as follows: 0.0–0.5 min, 95% A; 0.5–1.0 min, 40% A; 1.1–5.0 min, 95% A. The flow rate was set at 0.2 mL/min, with an injection volume of 10 µL, and the column temperature was maintained at 40 °C.

Data acquisition was carried out using electrospray ionization (ESI) in positive ion mode, employing multiple reaction monitoring (MRM). The ion source parameters were ion source temperature 150 °C, ion spray voltage 2.5 kV, desolvation temperature 350 °C, desolvation gas flow 800 L/h, and cone gas flow 50 L/h. Additional compound-specific parameters are listed in Table 1. Data acquisition and analysis were conducted on MassLynx 4.2 (Waters Corporation, Beverly, MA, USA).

### 2.6. Method Validation

The analytical method was validated in terms of linearity, limit of quantification (LOQ), trueness, and precision. Linearity was assessed by preparing a six-point calibration curve with standard solutions of the target biogenic amines in the concentration range of 0, 10, 50, 100, 150, 250 µg/L. The determination coefficient (R^2^) was calculated for each analyte. Quantification was performed using the standard addition approach, by fortifying sample aliquots with increasing concentrations of the analyte standard. Data processing was carried out using TargetLynx XS 4.2 software (Waters Corporation, Beverly, MA, USA), which enabled the automatic construction of the calibration curve while accounting for the endogenous amount already present in the matrix, estimated through the x-intercept of the regression line. The LOQs were fixed at the lowest validated concentration point.

To assess precision, intra-day repeatability (RSDr) was determined by calculating the coefficient of variation (CV%) from three replicates at each of three fortification levels (10, 100, and 250 µg/g). Inter-day reproducibility (RSDR) was evaluated by analyzing three replicates at each of the three levels over three separate days. Trueness was evaluated for each concentration level by spiking blank matrix samples after extraction with known amounts of analytes. All validation procedures were carried out considering acceptable CV ≤ 20% and trueness between −20% and +20%, following commonly accepted analytical standards for food contaminant analysis. 

## 3. Results and Discussion

### 3.1. Optimization of the Chromatographic Conditions

The optimization of the chromatographic method for detecting BAs was conducted through a series of trials aimed at achieving sufficient resolution, peak shape, and signal intensity for all target analytes.

**Figure 1:**
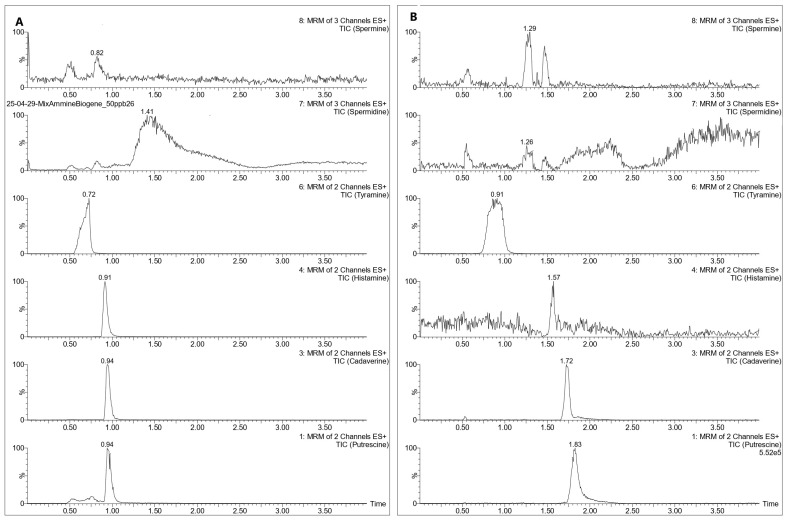
Chromatograms of a BAs solution (50 ng/g) using a Waters Acquity UPLC^®^ BEH HILIC (**A**), and a Waters Acquity UPLC^®^ BEH Amide (**B**).

An initial test was conducted on a Waters Acquity UPLC^®^ BEH HILIC (50 mm × 2.1 mm, 1.7 µm) column (Waters Corporation, Milford, MA, USA) column, with 20 mM ammonium formate in water acidified with 0.1% of formic acid (A) and acetonitrile (B) tested as mobile phases. Under these conditions, PUT, CAD, and HIS provided sufficiently satisfactory results, but TYR, SPD, and SPM exhibited poor peak shape and low response, compromising both resolution and detection sensitivity as shown in Figure 1A. Several gradient adjustments were attempted with the same column, but the issue persisted.

Given these limitations, a Waters Acquity UPLC^®^ BEH Amide (100 mm × 2.1 mm, 1.7 µm) column was tested to evaluate whether it could better retain analytes based on the method described by Lioupi et al. 2024 [18], whose instrumentation and mobile phases were comparable to those used in our setup. This method showed a deterioration of the chromatographic performance for HIS and failed to adequately separate SPM and SPD, which exhibited poor peak shape and low response, as shown in Figure 1B.

**Figure 2:**
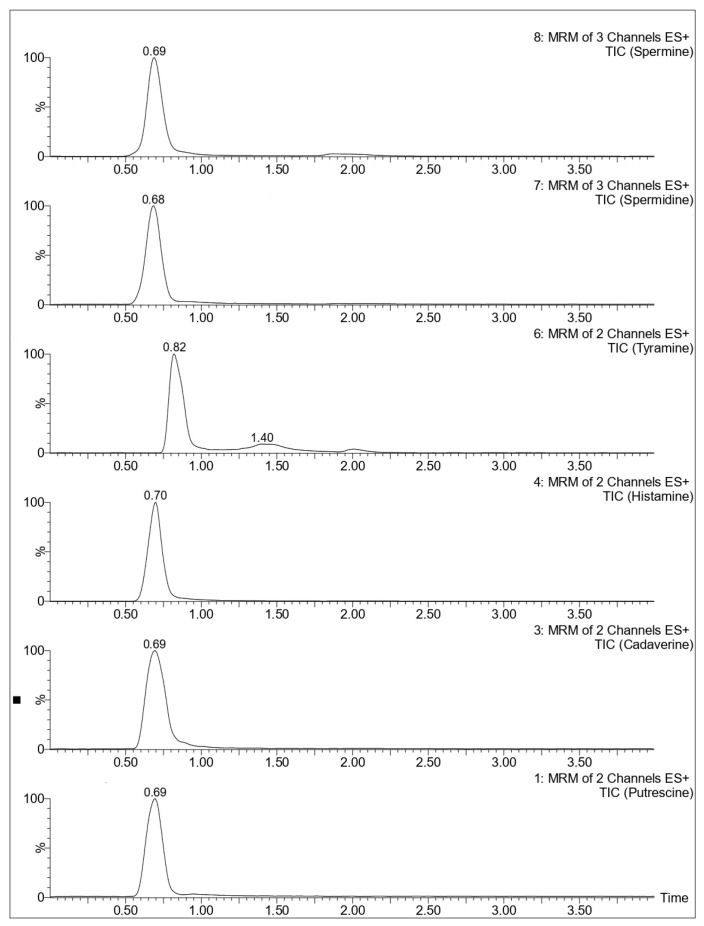
Chromatograms of a BAs solution (50 ng/g) using a Waters Acquity UPLC^®^ BEH C18 column.

Subsequently, the chromatographic conditions described by Xia et al. 2024 [17] were tested, as their method targeted the same panel of biogenic amines analyzed in the present study. A Waters Acquity UPLC^®^ BEH C18 column (50 mm × 2.1 mm, 1.7 µm; Waters Corporation, Milford, MA, USA) was used for the separation. The mobile phases consisted of water with 2 mM ammonium formate and 0.2% formic acid (A), and acetonitrile with 0.2% formic acid (B). These conditions provided the most satisfactory results in terms of peak shape, resolution, and signal intensity for all the analytes, as shown in Figure 2.

### 3.2. Optimization of BAs Extraction

To ensure sample integrity and prevent any alteration in the biogenic amine (BA) profile caused by microbial growth or analyte degradation, all meat samples were homogenized directly from the frozen state under controlled conditions and immediately aliquoted for subsequent extraction. The homogenization, extraction, and LC-MS/MS analysis were performed on the same day. This approach was adopted to minimize both the potential formation of BAs due to bacterial activity and the degradation or transformation of BAs in the extract.

To enhance the extraction procedure for all target biogenic amines, a series of preliminary tests were systematically performed. The optimization focused on two main parameters: the concentration of hydrochloric acid used for extraction and the type of filtration system employed. Different concentrations of HCl (0.1, 0.2, and 0.5 M) and volumes (3 and 4 mL) were tested to evaluate their impact on the extraction efficiency. Among the tested conditions, 4 mL of 0.5 M HCl proved to be the most effective. This combination facilitated a higher degree of solid precipitation and prevented the formation of gelatinous matrices, which can otherwise hinder phase separation and compromise extraction yields. Following centrifugation, samples extracted with 4 mL 0.5 M HCl consistently yielded a clearer supernatant and a higher recovery volume, which improved both the reproducibility of the protocol and the accuracy of quantification. Moreover, various filtering materials and filtration steps were compared to identify the most effective combination for obtaining a clear and analyte-rich extract while minimizing sample loss and matrix interference. Firstly, a double filtration step, before a paper filter, and subsequently a filtration with a syringe PFTE (0.22 μm) filter were tested. However, these filters often became clogged due to particulate matter, resulting in high backpressure, frequent leakage, and in some cases, the dislodging or bursting of the syringe during filtration. These issues were suspected to be a source of poor reproducibility observed when comparing results from samples extracted on different days. To address this, an additional centrifugation step was introduced after paper filtration to remove fine precipitates that could potentially be responsible for filter clogging.

The final protocol, selected based on its overall performance in terms of extraction efficiency, reproducibility, and compatibility with subsequent LC-MS/MS analysis, proved to be less time-consuming, relatively inexpensive, with minimal material consumption, and did not require derivatization. Compared to other methods reported in the literature [3,5,8,17], it appears simpler and user-friendly, thus supporting the monitoring of biogenic amines in meat.

### 3.3. Method Validation

The method for quantifying biogenic amines was validated over three separate days using spiked samples at three concentration levels (10,100, 250 µg/g), applying the standard addition approach due to the natural presence of the analytes in the matrix. Details are provided in Table 2 and Table 3. 

**Figure 3:**
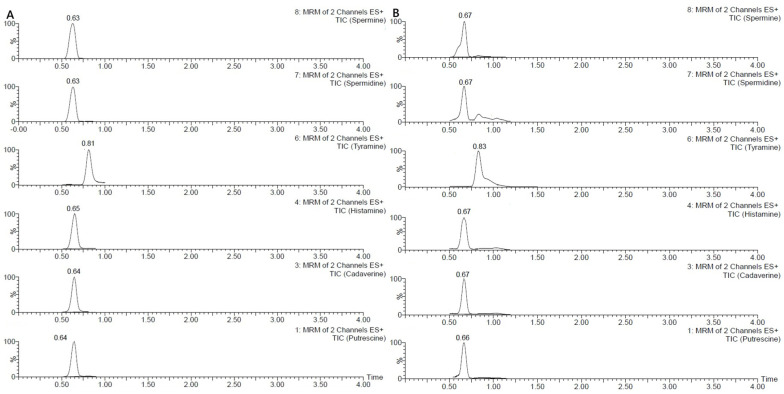
Chromatograms of BAs at LOQ (**A**), and in sample 5 (**B**).

The method showed good trueness (within ±20%) and acceptable precision, with RSDr% and RSDR% values ≤ 20%. However, for two analytes, particularly SPD and SPM, results occasionally approached the limits of the established acceptance criteria. In one instance, for SPD at the lowest concentration on day three, the RSDr% reached 25%. The LOQs were established at 10 µg/g for all analytes (Figure 3A). Matrix effects were considered negligible due to the high dilution factor applied during sample preparation. Specifically, the extract was diluted 1:500 before injection, resulting in a very low residual matrix content in the final solution. Overall, the method proved to be accurate for the determination of biogenic amines in the tested matrix.

### 3.4. Application to Real Samples

The validated method was applied to ten meat samples collected from the market of raw beef to evaluate its practical applicability. During meat storage, microbial and enzymatic proteolysis lead to the degradation of proteins into peptides and subsequently into free amino acids, which serve as precursors for the microbial decarboxylation reactions responsible for the formation of other BAs, such as PUT, CAD, HIS, and TYR [1].

Among the analytes investigated, PUT, TYR, and SPD were the most frequently detected, while CAD and HIS were generally found at lower levels or below the LOQ. Results are reported in Table 4. Notably, samples 5 (Figure 3B), 6, and 9 showed the highest concentrations of PUT, CAD, HIS, and TYR, indicating a more intense microbial activity, which may be attributed to improper handling or prolonged storage. In contrast, other samples (e.g., 1, 2, 3, 4, 7, 8, and 10) showed only low levels of a few amines, indicating minimal degradation or fermentation. These results confirm the method’s suitability for routine monitoring of biogenic amines in complex meat matrices across a range of quality conditions. Our results are in line with those reported in the literature, reviewed by Schirone et al. 2022 [1].

Importantly, the detected concentrations of HIS and TYR were substantially below toxicologically relevant thresholds for humans. According to current literature, HIS intoxication in healthy individuals is unlikely at exposure levels below 25–50 mg per meal, while symptoms may occur in HIS-intolerant individuals at doses as low as 5–10 mg [2]. Similarly, TYR toxicity becomes a concern primarily in individuals treated with monoamine oxidase inhibitors (MAOIs), where safe exposure levels can range from 6 mg to 50 mg per meal depending on the drug generation [2,24]. In healthy individuals, no adverse health effects were observed from up to 600 mg of tyramine per person per meal [2]. The maximum levels found in our samples (17.9 µg/g for HIS and 66.5 µg/g for TYR) correspond to 1.7 mg and 6.5 mg per 100 g of serving portion, respectively—values lower than those associated with adverse effects in non-sensitive populations. Nevertheless, sample 5, with a TYR content of 6.5 mg/100 g, could represent a risk for MAOIs-treated individuals.

In addition to individual amine concentrations, BAI was calculated for each sample using the formula [6]:

$$BAI=\frac{\left(HIS\;+\;PUT\;+\;CAD\right)}{\left(1+SPD+SPM\right)}$$

with concentrations expressed in µg/g.

To facilitate index calculation, a middle-bound approach was applied by assigning a value equal to half the LOQ to concentrations of BAs below the limit of quantification. The BAI is regarded as a reliable indicator of hygienic quality: values below 1 indicate good quality, while values above 10 reflect poor microbiological status [6]. In this study, BAI values ranged from 0.5 (sample 3) to 4.2 (sample 6). Most samples showed BAI values below 1, suggesting acceptable quality and limited microbial degradation. However, samples 5, 6, and 9 showed modest BAI values (ranging from 3.3 to 4.2), indicating potential quality deterioration or early spoilage. Nevertheless, none of the samples approached the threshold indicative of very poor quality (BAI > 10), highlighting its advanced degradation stage. The observed variability among samples may be attributed to several factors, including differences in initial microbial contamination, hygiene during processing, and storage conditions. However, these findings underline the relevance of BAI as a rapid screening tool for assessing the freshness and safety of meat products.

## 4. Conclusions

A rapid, robust, and reproducible LC–MS/MS method was developed and validated for the simultaneous quantification of biogenic amines in meat products. The protocol was optimized to enhance extraction efficiency and chromatographic resolution, particularly for structurally similar polyamines. The method showed good analytical performance in terms of sensitivity, trueness, and precision, and was successfully applied to real meat samples. Among the detected amines, PUT, TYR, and SPM were the most prevalent. Despite the detection of HIS and TYR in some samples, all concentrations remained below toxicologically relevant thresholds for the general population. Only one sample (sample 5) approached levels that may pose a risk to individuals under MAOI treatment.

The calculation of the BAI further supported the assessment of product quality, identifying early spoilage in selected samples. Overall, the method provides a valuable tool for routine monitoring of BAs in meat, supporting both food quality control and consumer safety.

## Figures and Tables

**Table 1:** MS/MS conditions.

Compound	Precursor (m/z)	Fragment 1 (m/z)	Fragment 2 (m/z)	Collision Energy (V)	Cone Voltage (kV)
HIS	112.0	95.0	67.9	10, 30	2.5
CAD	102.9	86.0	69.0	10, 13	2.5
PUT	89.0	72.0	30.0	10, 25	2.5
SPM	203.1	129.1	112.0	10, 20	2.5
SPD	146.0	71.9	112.0	15, 15	2.5
TYR	138.1	121.0	76.9	10, 25	2.5

Note: Fragment 1 was used for quantification.

**Table 2:** Validation parameters at three spike levels ^a^.

Compound	Trueness (Bias%)				RSD_r_%			RSD_R_%
	Day 1	Day 2	Day 3	Interday	Day 1	Day 2	Day 3	
PUT	−2; +7; +6	−17; −9; −11	−17; −7; −9	−12; −3; −5	8; 3; 5	11; 4; 2	9; 9; 5	12; 9; 9
CAD	+3; −4; +2	−9; −4; +1	+16; +3; +19	+3; −1; +7	7; 1; 2	14; 6; 0	8; 5; 10	13; 5; 10
HIS	−1; −4; +1	−6; −9; −3	−1; +2; +4	−3; −3; +1	3; 2; 4	12; 3; 4	14; 10; 7	10; 7; 6
TYR	+11; −3; +10	−10; −15; +14	−11; −18; 0	−3; −12; +8	7; 1; 6	8; 6; 3	5; 9; 14	13; 9; 10
SPD	−4; −5; +2	+20; +20; +20	+9; +19; +8	+8; +11; +10	14; 5; 11	15; 9; 1	25; 4; 7	19; 12; 9
SPM	+7; −12; −7	+20; +4; +3	−11; +20; +15	+5; +4; +4	11; 19; 13	4; 13; 4	15; 8; 16	16; 17; 14

^a^ 10, 100, and 250 µg/g.

**Table 3:** BAs calibration curve parameters and LOQs.

Compound	R^2^	Linear Equation	LOQ (µg/g)
PUT	0.996	y = 0.033x + 0.063	10
CAD	0.997	y = 0.061x − 0.036	10
HIS	0.998	y = 0.065x − 0.028	10
TYR	0.996	y = 0.004x + 0.005	10
SPD	0.996	y = 0.616x + 1.60	10
SPM	0.996	y = 0.672x + 0.545	10

R^2^: correlation coefficient.

**Table 4:** Real samples analysis results.

Sample	PUT	CAD	HIS	TYR	SPD	SPM	BAI ^a^
1	<LOQ	<LOQ	<LOQ	<LOQ	<LOQ	<LOQ	1.3
2	<LOQ	<LOQ	<LOQ	<LOQ	<LOQ	12.7	0.8
3	<LOQ	<LOQ	<LOQ	<LOQ	12.7	16.3	0.5
4	12.6	<LOQ	<LOQ	10.6	14.7	21.8	0.6
5	29.9	<LOQ	<LOQ	66.5	<LOQ	<LOQ	3.3
6	18.8	13.6	17.9	23.8	<LOQ	<LOQ	4.2
7	<LOQ	<LOQ	<LOQ	25.5	<LOQ	<LOQ	1.3
8	<LOQ	<LOQ	<LOQ	<LOQ	<LOQ	<LOQ	1.3
9	30.8	<LOQ	<LOQ	50.6	<LOQ	<LOQ	3.4
10	<LOQ	<LOQ	<LOQ	<LOQ	10.4	10.1	0.7

^a^ Biogenic Amines Index. Results are expressed in µg/g.

## Data Availability

Data available on request from the authors.
